# Cemented implantation of a dual mobility cup in an existing acetabular cup to prevent recurrent dislocation after total hip replacement

**DOI:** 10.1007/s00132-025-04733-5

**Published:** 2025-11-03

**Authors:** E. Saleh, S. Yacoub, C. Pempe, A. Roth, M. Ghanem

**Affiliations:** 1 Department of Orthopedics, Traumatology and Plastic Surgery, University Hospital Leipzig, Liebigstraße 18, 04103 Leipzig, Germany; 2 Department of Physical Therapy and Rehabilitation, University Hospital Leipzig, Leipzig, Germany

**Keywords:** Dual mobility cup (DMC), Cup-in-cup technique, DMC direct in bone technique, Dislocation, Instability, DMC, Cup-in-Cup-Technik, Dual-Mobility-Cup-Technik direkt in den Knochen, Dislokation, Instabilität

## Abstract

**Background:**

Recurrent hip dislocation is a common complication following total hip arthroplasty (THA). The dual mobility cup (DMC) technique was introduced to reduce the risk of dislocation. Cementing DMC in a fixed acetabular cup may be a good alternative to the usual approach of implanting it directly into bone. We aimed to evaluate the outcome of DMC in preventing recurrent THA dislocation. In addition, we examined differences in clinical outcomes between the two techniques: cementation into a pre-existing acetabular cup versus direct implantation into bone.

**Methods:**

The sample comprised 20 patients who underwent surgery using the DMC technique between 2014 and 2023. The primary endpoint was time to the occurrence of postoperative revision operation for any reason, assessed by the Kaplan-Meier survival analysis with a significance threshold of *p*  < 0.05.

**Results:**

This retrospective comparative study included a total of 20 patients: 10 with a cemented DMC in a fixed acetabular cup and 10 with cementation directly in bone. Each patient experienced at least one hip dislocation following THA. The mean age of the patients was 77.65 years. The mean follow-up duration was 9.91 ± 17.01 months. The average number of preoperative dislocations was 2.90 ± 1.97. A comparison between the outcomes of the two techniques showed no significant differences.

**Conclusion:**

Cemented DMC has shown favorable results in recent years, particularly in reducing the dislocation rate following revision THA in older patients. This makes it a valuable option to prevent more extensive surgery. Cementation in a fixed acetabular cup showed similar outcomes to the conventional approach, with no noticeable drawbacks.

**Graphic abstract:**

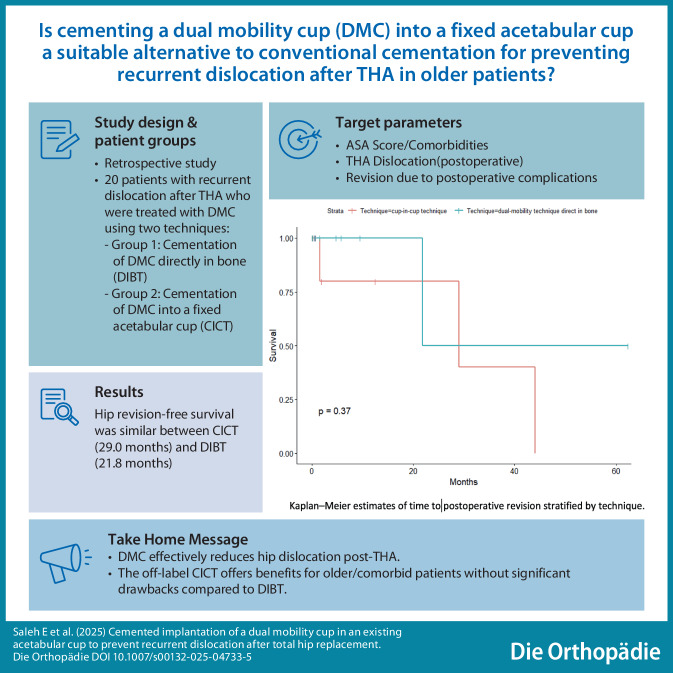

## Abstract (de)

**Hintergrund:**

Rezidivierende Hüftluxationen sind eine häufige Komplikation nach einer Hüfttotalendoprothese (HTEP). Die Technik der sog. Dual-Mobility-Cup (DMC) wurde eingeführt, um das Risiko einer Luxation zu senken. Die Zementierung der DMC in eine vorhandene Hüftgelenkspfanne kann eine gute Alternative zum üblichen Ansatz der direkten Implantation in den Knochen sein. Unser Ziel war es, das Ergebnis der DMC bei der Verhinderung einer erneuten HTEP-Luxation zu bewerten. Darüber hinaus untersuchten wir die Unterschiede in den klinischen Ergebnissen zwischen den beiden Techniken: Zementierung in eine bereits vorhandene Hüftpfanne und direkte Implantation in den Knochen.

**Methoden:**

Die Stichprobe umfasste 20 Patienten, die sich zwischen 2014 und 2023 einem chirurgischen Eingriff mit der DMC-Technik unterzogen. Der primäre Endpunkt war die Zeit bis zum Auftreten einer postoperativen Revisionsoperation aus jedwedem Grund, bewertet mittels Kaplan-Meier-Überlebensanalyse mit einer Signifikanzschwelle von *p* < 0,05.

**Ergebnisse:**

An dieser retrospektiven Vergleichsstudie nahmen insgesamt 20 Patienten teil: 10 mit einer zementierten DMC in einer festen Hüftpfanne und 10 mit einer Zementierung direkt im Knochen. Jeder Patient hatte mindestens eine Hüftluxation nach einer HTEP erlitten. Das Durchschnittsalter der Patienten betrug 77,65 Jahre. Die mittlere Nachbeobachtungszeit betrug 9,91 ± 17,01 Monate. Die durchschnittliche Anzahl der präoperativen Luxationen betrug 2,90 ± 1,97. Ein Vergleich zwischen den Ergebnissen der beiden Techniken zeigte keine signifikanten Unterschiede.

**Schlussfolgerung:**

Die zementierte DMC hat in den letzten Jahren positive Ergebnisse gezeigt, insbesondere bei der Reduzierung der Luxationsrate nach Revisions-HTEP bei älteren Patienten. Dies macht sie zu einer wertvollen Option zur Vermeidung größerer Operationen. Die Zementierung in einer vorhandenen festen Hüftgelenkspfanne zeigte ähnliche Ergebnisse wie der konventionelle Ansatz, ohne nennenswerte Nachteile.

## Introduction

Total hip arthroplasty (THA) is one of the most commonly used and successful surgical procedures, significantly improving the quality of life in the aging population. It is particularly beneficial for those suffering from coxarthrosis or a fractured neck of the femur [1]. Hip dislocation is a frequent postoperative complication [2] and a cause of higher morbidity, especially among revision THA operations [3, 4]. Other complications include fractures (18.2%) and surgical site infections (17.2%) [5]. The hip dislocation rate after revision THA is higher in comparison to primary THA and is considered the main cause for further revision THA [6, 7]. The number of THA revision operations is expected to increase by 137% by 2030 [8]. A study by de Cano et al. reported a 35% dislocation rate after THA revision due to instability [9]. In a meta-analysis of 125 studies Kunutsor et al. reported that the incidence rate of instability of the primary THA was 7% and it was 25% higher after revision THA [10].

The main risk factors for THA dislocation can be patient-related or implant-related in addition to the surgeon’s experience. Patient-related factors include previous hip operations, advanced age and lack of abductor muscular support [11–13]. Furthermore, Kunutsor et al. reported that white race, high American Society of Anesthesiologists (ASA) score, high body mass index (BMI) > 30 kg/m^2^, low social level, drug use as well as psychiatric and neurological disorders increase the risk of dislocation [10]. Implant-associated factors include the size of the femoral head, the size of the acetabular cup and the degree of femoral neck offset [14]. For example, Jameson et al. reviewed 247,546 procedures and reported a decline in postoperative dislocation incidence with the use of larger femoral heads [15].

The dual mobility cup (DMC) was pioneered in the 1970s by Bousquet and Rambert [16]. The DMC features a bipolar component that moves inside a fixed large diameter metal shell and it provides a better range of motion (ROM) before impingement. Therefore, it is expected to provide better stability with a lower rate of dislocation [17, 18]. Civinini et al., Jakobsen et al. and Van Heumen et al. reported successful clinical results for treating recurrent THA dislocation using DMC [19–21]. In a study with a 22-year follow-up of 240 hips using DMC, recurrent THA dislocations occurred only in 7 cases (0.2%), which was nonsignificant [22]. Hamadouche et al. also showed successful restoration of hip stability in 96% of 51 patients using DMC during revision THA [23]. The removal of well-fixed components, especially cemented acetabular shells, during revision THA can be highly challenging. This is mainly due to an increased risk of complications such as longer operation times, bone damage, greater blood loss and a higher incidence of infections [24]. One of the DMC approaches was described as “off-label cementation of DMC into a fixed metal shell” [25, 26], which we refer to in our work as the cup-in-cup technique (CICT). This approach was initially used to revise failed THA, particularly in older patients with comorbidities and higher ASA scores, with the aim of reducing complications [26, 27].

Herein we present two approaches for using DMC in THA revision surgery, the previously mentioned CICT versus the implantation directly into bone, which we referred to as direct-in-bone technique (DIBT). We aimed to assess the difference using DMC between both techniques in terms of stability and rate of dislocation.

## Patients and methods

Between September 2014 and December 2023, we retrospectively reviewed 20 patients who suffered from recurrent postoperative hip dislocation after primary or revision THA. We included patients who were surgically treated with cementing CICT or DIBT at our center. Patient-related factors for choosing CICT were: (1) recurrent dislocation in which removal of the acetabular component may cause bone loss and/or bleeding, (2) failure of prior surgical hip stabilization operations, or (3) multimorbid situations requiring shorter operation duration to avoid complications. Whenever CICT was not possible due to instability of the pre-existing acetabular cup, we used DIBT. We preferred using DMC rather than constrained liners in those patients because of the increased failure rate and loosening with constrained implants [26, 28, 29].

Baseline characteristics including age, sex, BMI, ASA, date of the primary hip implantation, operation site, previous operations at the same hip joint, number of hip dislocation occurrences before surgery and onset of hip dislocation following the operation were derived from the patients’ electronic records. We also reviewed the digital operative records for information on the duration of surgery, type of implant and complications during the surgery to evaluate the invasiveness of the intervention.

Our main outcome was time (months) to postoperative revision operation following DMC, which was mainly due to postoperative hip dislocation. Secondary outcomes included surgery duration, postoperative complications, capability of mobility using the modified Harris Hip Score (mHHS) [30] as well as the Barthel Index (BI) [31].

Intraoperatively, histological and microbiological samples were taken. All patients were initially investigated with X‑rays in which we planned the expected implant size before the operations.

The patients were examined preoperatively, postoperatively and at discharge and 19 patients were alive at the final follow-up.

The patients were classified into 2 groups based on the surgical technique. Group 1 (10 patients) underwent CICT involving either “cementation of DMC into a preexisting acetabular cup” (7 patients) or in Burch Schneider reinforcement ring (BSR, 3 patients). Group 2 (10 patients) received DIBT for the treatment of hip dislocation. Contrary to De Cano and Trias [9], we considered BSR to fall under CICT to differentiate easily between the groups of interest and to highlight the practical impact on the patients with each technique.

### Surgery

The operation was performed with the patient under general anesthesia for all the patients. The anterior lateral approach was used in all patients, following previous THA operations. After hip exposure, the femoral head and polyethylene (PE) liner were removed. The acetabular cup as well as the femoral shaft were tested for stability. The surgeon checked whether the acetabular cup was loose or not and chose the appropriate technique (either CICT or DIBT). For DMC implantation as CICT, the screws (if present) were removed and the acetabular cup direction was assessed to determine the better direction for the DMC. The DMC components were determined according to the previous planning on X‑ray imaging. Then, the DMC was placed and cemented with the desired inclination. The cement hardened in 8–10 min and was then irrigated and trial components were used to verify its size and stability. The definite metal head and liner were inserted and checked for impingement or dislocation. Vancomycin powder was applied before closure of the wound in 30% of patients [32] and the wound was closed layer by layer.

The hip instability or subluxation after the operation was considered as a form of dislocation. In this research, instability and/or subluxation was defined by detecting any abnormal movement of the hip joint coupled with pain or any audible clunk, or any sign of instability in X‑ray images.

### Statistical analysis

Continuous variables were presented as mean and standard deviation (SD) while categorical variables as frequencies and percentages. Statistical significance was evaluated using the independent t‑test for continuous variables when assumptions of normality were met, or nonparametric tests otherwise. The χ^2^-test was used for categorical variables as appropriate. Survival analysis was performed via the Kaplan-Meier method. A *p*-value of ≤ 0.05 was considered statistically significant. We conducted statistical analyses via the Statistical Package for the Social Sciences (SPSS) software (version 26.0) (IBM, Armonk, NY, USA).

## Results

### Baseline characteristics

A total of 20 patients participated in this study. Half of the patients underwent CICT and the other half underwent DIBT for the treatment of recurrent hip dislocation after THA. The study included 15 women and 5 men with a mean age at the time of the operation of 77.65 ± 13.10 years (range 34–95 years) and 1 patient died shortly after the operation as she had multiple comorbidities. The mean follow-up duration was 9.91 ± 17.01 months. The mean BMI was 26.36 kg/m^2^ (range 19.2–41.4 kg/m^2^). Regarding ASA scores, the mean was 2.6, with 8 patients (40%) having ASA II and 12 patients (60%) having ASA III.

All revision operations were performed for THA dislocation. Among the patients, 16 patients (80%) had recurrent dislocation, 2 patients (10%) had dislocation with femoral loosening, 1 patient (5%) had a dislocation with periprosthetic fracture, and 1 patient (5%) had a dislocation with acetabular fracture. The mean number of preoperative dislocations was 2.90 ± 1.97. The most common secondary diagnoses at the time of surgery were osteoporosis (12 patients) and hypertension (11 patients). At the time of the revision hip operation, all patients had a history of at least 1 previous hip operation, with a mean of 2.30 ± 1.95 (range 1–7). The mean duration of hospitalization was 15.75 ± 8.06 days, 12 surgeries (60%) were performed on the right hip, while the remaining surgeries (40%) were performed on the left hip (Table 1).

**Table 1:** Baseline characteristics

Baseline	
*Age at surgery (years) ±SD (range)*	77.65 ± 13.10 (34–95)
*ASA score*	2.6 ± 0.5
ASA 2	8/20 (40.0%)
ASA 3	12/20 (60.0%)
*Diagnosis*	
Dislocation with acetabular fracture	1 (5.0%)
Dislocation with acetabular loosening	2 (10.0%)
Dislocation with periprosthetic femoral fracture	1 (5.0%)
Recurrent dislocation	16 (80.0%)
*Surgery duration (min)*	96.55 ± 45.90
*Site of operation*	
Left	8 (40.0%)
Right	12 (60.0%)
*Technique type*	
Cup-in-cup	10 (50.0%)
Dual-mobility cup directly in bone	10 (50.0%)
*Follow-up duration (months)*	9.91 ± 17.01
*Hospital stay (days)*	15.75 ± 8.06
*Patient mobility before the operation*	
Able to walk only using 2 forearm crutches (limping)	2 (10.0%)
Impaired ability to walk and stand due to pain	1 (5.0%)
Mobile using a wheelchair	2 (10.0%)
Not mobile due to hip dislocation	13 (65.0%)
Not mobile due to periprosthetic femoral fracture	1 (5.0%)
Not mobile in ICU	1 (5.0%)
*Sex*	
Female	15 (75.0%)
Male	5 (25.0%)

*ASA* American Society of Anesthesiologists, *ICU* intensive care unit

The distribution of the number of revision operations was as follows: 2 revision surgeries among 9 patients, 3 among 4 patients, 4 among 3 patients, 5 among 3 patients and 8 surgeries in 1 patient. One patient had a preoperative infection that was successfully treated at the time of surgery. The revision operation was performed at an average of 8 years (range 1 month–25 years) after the primary THA operation.

The mean number of previous revision operations in the same hip joint was 2.30 ± 1.55. After the operation, the mean number of hip joint dislocations was 0.40 ± 0.97. The size of the cups used in the surgeries had a mean of 51.25 ± 4.64 mm. All patients did not require wearing an antidislocation brace or bandage after the operation (Table 2). Intraoperative complications, including fractures, cardiovascular events, pulmonary embolism and death did not occur in any of the patients.

**Table 2:** Output table illustrates the comparative analysis of CICT and DIB

Variable	Levels	CICT (*n* = 10)	DIBT (*n* = 10)	*P*-value
Age of the patient at the time of the operation (years mean ± SD)	–	79.10 ± 7.71	76.2 ± 19.19	–
ASA score at the time of the operation (%)	ASA 2	50%	30%	–
	ASA 3	50%	70%	
Previous operations in the same hip joint (%)	Yes	10 (100.0%)	10 (100.0%)	NA
Number of previous operations in the same hip joint (mean ± SD)	–	2.30 ± 1.95	2.30 ± 1.16	1
Number of previous revision operations in the same hip joint (%)	1 operation	5 (50.0%)	3 (30.0%)	0.669
	2 operations	2 (20.0%)	3 (30.0%)	–
	3 operations	1 (10.0%)	2 (20.0%)	–
	4 operations	1 (10.0%)	2 (20.0%)	–
	7 operations	1 (10.0%)	0 (0.0%)	–
Patients had hip dislocations before the operation (%)	Yes	10 (100.0%)	10 (100.0%)	NA
Number of hip dislocations before the operation (mean ± SD)	–	2.40 ± 2.17	3.40 ± 1.78	0.274
Number of hip dislocations before the operation (%)	0 operation	1 (10.0%)	0 (0.0%)	0.077
	1 operation	4 (40.0%)	1 (10.0%)	–
	2 operations	2 (20.0%)	2 (20.0%)	–
	3 operations	0 (0.0%)	4 (40.0%)	–
	4 operations	1 (10.0%)	0 (0.0%)	–
	5 operations	0 (0.0%)	2 (20.0%)	–
	6 operations	2 (20.0%)	0 (0.0%)	–
	7 operations	0 (0.0%)	1 (10.0%)	–
Patients had hip dislocation after the operation (%)	No	8 (80.0%)	8 (80.0%)	1
	Yes	2 (20.0%)	2 (20.0%)	–
Number of hip dislocations after the operation (mean ± SD)	–	0.40 ± 0.97	0.30 ± 0.67	0.791
Number of hip dislocations after the operation (%)	0 operation	8 (80.0%)	8 (80.0%)	0.572
	1 operation	1 (10.0%)	1 (10.0%)	–
	2 operations	0 (0.0%)	1 (10.0%)	–
	3 operations	1 (10.0%)	0 (0.0%)	–
Wearing an antidislocation brace (%)	No	10 (100.0%)	10 (100.0%)	NA
Wearing an antidislocation bandage (%)	No	10 (100.0%)	10 (100.0%)	NA
*Intraoperative complications*				
Fracture (%)	No	10 (100.0%)	10 (100.0%)	NA
Cardiovascular event (%)	No	10 (100.0%)	10 (100.0%)	NA
Pulmonary embolism (%)	No	10 (100.0%)	10 (100.0%)	NA
Death (%)	No	10 (100.0%)	10 (100.0%)	NA
*Postoperative complications*				
Renewed dislocation (%)	No	8 (80.0%)	8 (80.0%)	1
	Yes	2 (20.0%)	2 (20.0%)	–
Loosening (%)	No	9 (90.0%)	9 (90.0%)	1
	Yes	1 (10.0%)	1 (10.0%)	–
Liner wear (%)	No	9 (90.0%)	10 (100.0%)	1
	Yes	1 (10.0%)	0 (0.0%)	–
Had postoperative revision operations (%)	No	7 (70.0%)	9 (90.0%)	0.576
	Yes	3 (30.0%)	1 (10.0%)	–
Postoperative revision operations (mean ± SD)	–	0.40 ± 0.70	0.20 ± 0.63	0.511
Postoperative revision operations (%)	0 operation	7 (70.0%)	9 (90.0%)	0.325
	1 operation	2 (20.0%)	0 (0.0%)	–
	2 operations	1 (10.0%)	1 (10.0%)	–
Postoperative complication: hip infection (%)	No	8 (80.0%)	8 (80.0%)	1
	Yes	2 (20.0%)	2 (20.0%)	–
Postoperative complication: wound infection (%)	No	9 (90.0%)	10 (100.0%)	1
	Yes	1 (10.0%)	0 (0.0%)	–
Internal in-hospital postoperative complication: pulmonary embolism (%)	No	9 (90.0%)	10 (100.0%)	1
	Yes	1 (10.0%)	0 (0.0%)	–
Internal in-hospital complication: pneumonia (%)	No	10 (100.0%)	9 (90.0%)	1
	Yes	0 (0.0%)	1 (10.0%)	–
Internal in-hospital complication: urinary infection (%)	No	8 (80.0%)	8 (80.0%)	1
	Yes	2 (20.0%)	2 (20.0%)	–
Internal in-hospital complication: cardiovascular (%)	No	9 (90.0%)	9 (90.0%)	1
	Yes	1 (10.0%)	1 (10.0%)	–
Internal in-hospital complication: death (%)	No	10 (100.0%)	9 (90.0%)	1
	Yes	0 (0.0%)	1 (10.0%)	–
Ability to walk at discharge (%)	Mobile using 2 forearm crutches under pain-adapted full load	4 (40.0%)	3 (30.0%)	0.214
	Mobile using 2 forearm crutches with 20 kg partial load	3 (30.0%)	2 (20.0%)	–
	Mobile using rollator	0 (0.0%)	3 (30.0%)	–
	Patient at the walking bench with pain-adapted full weight bearing with support for a few steps in the room mobile	2 (20.0%)	0 (0.0%)	–
	To the edge of the bed and to the walking bench 2–3 steps	1 (10.0%)	0 (0.0%)	–
	Mobile using wheelchair	0 (0.0%)	1 (10.0%)	–
	Not mobile in ICU	0 (0.0%)	1 (10.0%)	–
Barthel index postoperatively (mean ± SD)	–	81.00 ± 17.29	71.11 ± 25.95	0.337
Modified Harris hip score mHHS after the operation (mean ± SD)	–	60.89 ± 14.65	48.67 ± 23.07	0.198

*ASA *American Society of Anesthesiologists, *CICT* cup in cup technique, *DIBT* direkt in bone technique, *ICU* intensive care unit, *SD* standard deviation

Data are presented as mean ± SD or number (percentage). Categorical variables were analyzed via the χ^2^ test or Fisher’s exact test, and the independent t‑test test was used for continuous variables. *P*-values ≤ 0.05 were considered statistically significant.

### Outcome

A comparison of the two surgical techniques revealed that the mean operation duration for CICT was 102.80 ± 57.51 min, whereas that for DIBT was 91.42 ± 31.31 min (*p* = 0.657). The mean follow-up duration for CICT was 9.10 ± 15.33 months and for DIBT it was 11.74 ± 20.23 months (*p* = 0.75). Assessing the mHHS at the last follow-up after the operation revealed a mean score of 60.89 for the CICT group and 48.67 for the DIBT group (*p* = 0.198) (Table 2). In terms of postoperative walking ability, both groups had improved outcomes compared to the preoperative conditions. Following the operation, all patients who underwent CICT were mobile, whereas 80% of those who received DIBT were mobile. In practice, there was no significant difference in mobility outcomes between the CICT and DIBT groups (*p* = 0.214) (Table 2).

We found that two patients in each group experienced postoperative dislocation once. There was no difference in the incidence of postoperative dislocations between the two techniques (*p* = 1). Postoperative complications, including renewed dislocation or cup loosening, were not significantly different between the two techniques. Postoperative assessment of the Barthel index at discharge revealed a mean score of 81.00 for the CICT group and 71.11 for the DIBT group (*p* = 0.337) (Table 2).

In conclusion, this study encompassed 20 patients and compared the CICT with the usual DIBT for the treatment of hip dislocation. The results demonstrated no significant differences in patient demographics, operative outcomes, postoperative complications, or functional outcomes between the two surgical techniques in this patient population.

**Fig. 1:**
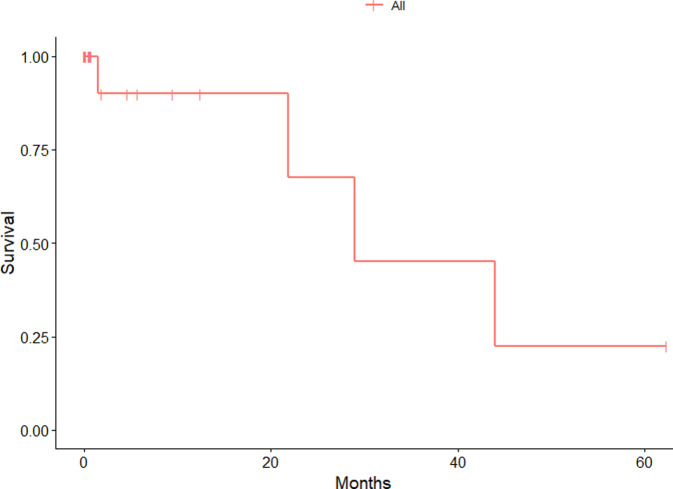
Kaplan-Meier estimates of the time to postoperative revision for all techniques

**Fig. 2:**
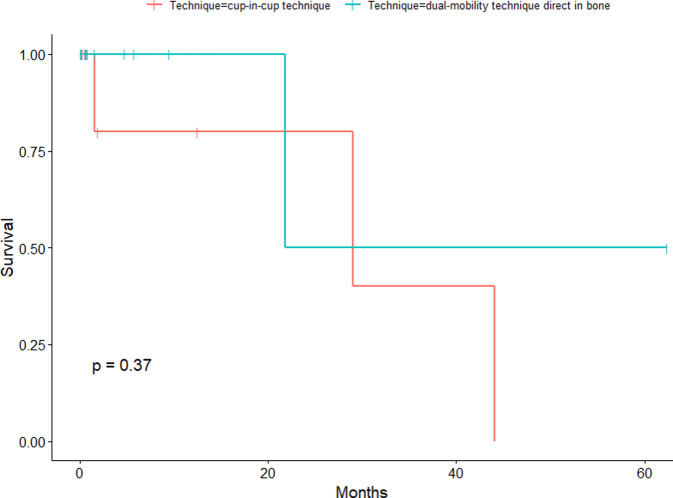
Kaplan-Meier estimates of time to postoperative revision stratified by technique

The endpoint of the study was time to postoperative revision of the hip due to any cause after implantation of DMC. All patients with an available final follow-up were included in the survival analysis (Fig. 1). A comparison of the cup survival time between the 2 groups via the Kaplan-Meier method revealed a median survival time of 29.0 months for CICT patients and 21.8 months for DIBT patients. The log-rank test revealed no significant difference in the time to postoperative revision between the two techniques (*P* = 0.37) (Fig. 2).

## Discussion

This study examined 20 patients who underwent revision THA using cemented DMC in our center from 2014 to 2023. These patients suffered from recurrent dislocation after THA. We focused on the results of DMC in reducing postoperative recurrent hip dislocation and determined whether there was a difference between the 2 implantation techniques (CICT or DIBT).

**Fig. 3:**
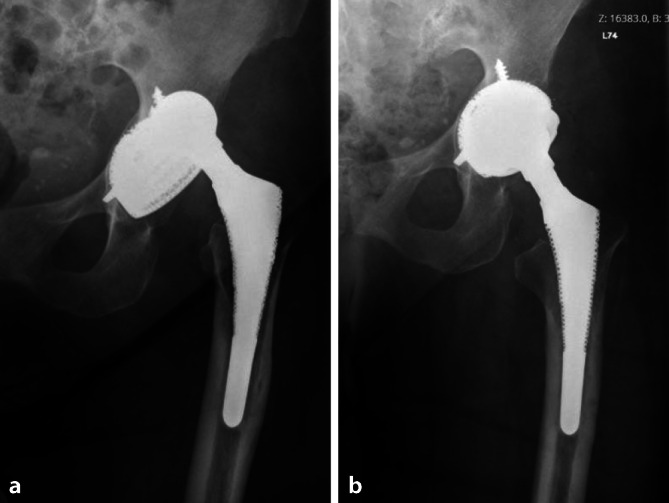
Case of a 79-year-old man with septic loosening and dislocation after total hip arthroplasty (**a**). Placement of cemented DMC into the existing well-fixed metal shell “CICT” (**b**)

**Fig. 4:**
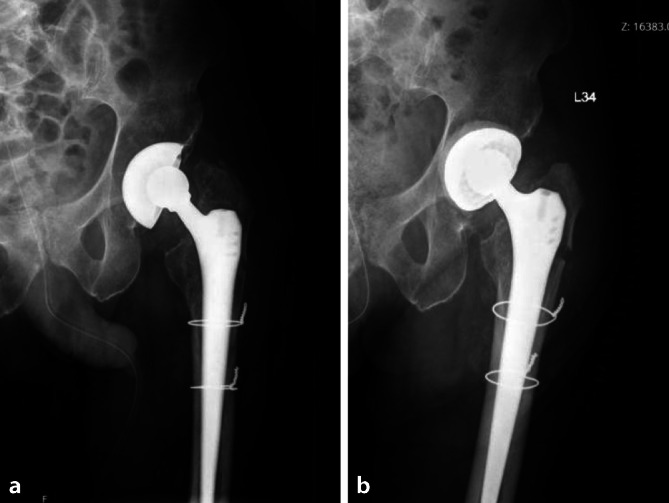
Case of a 75-year-old man operated on for multiple recurrent THA dislocations (**a**), with replacement of prosthetic components and placement of cemented DMC directly to the bone “DIBT” (**b**)

The type of technique differed according to the indications for the operation, the acetabular bone condition and the state of the pre-existing acetabular cup. If the pre-existing implant was stable and of sufficient size (48 mm or more), the surgeon opted for CICT to minimize bone damage and blood loss (Fig. 3). If the implant was loose, it was removed and DIBT was chosen (Fig. 4).

Our overall goal was to compare the outcomes between CICT and DIBT based on the technique (Table 2). Secondly, we compared the clinical outcomes in our study after the use of DMC in reducing the dislocation rate after revision THA with the findings of previous studies (Table 3). Finally, we compared the outcomes of 10 patients who underwent DMC as CICT (group 1) with those in previous related studies (Table 4).

**Table 3:** Comparison of several clinical studies on the DMC (regardless of the technique used) [33]

Author	Year	Number of patients (only cemented DMC)	Surgical approach	Mean follow up; months, (range), <SD>	Aseptic loosening	Dislocation	Infection	Revision	Cup survivorship (%)
Stambough [34]	2017	8	Posterior/posterolateral	34.8 (24–63.6)	0	2	1	2	85
Hipfl [35]	2018	15	–	47 (25–84)	0	1	2	–	89
Assi [36]	2019	16	Posterior/posterolateral	72.9 <± 40.5>	0	0	0	0	98.8
Wheelton [37]	2019	54	–	22.8 (6–60)	0	0	1	1	95.8
Schmidt [38]	2020	59	Posterior/posterolateral	24 (12–141.6)	–	4	–	13	95.6
Bozon [39]	2021	23	Posterior/posterolateral	108 <± 12>	2	1	3	2	87
Saleh^1^	2023	20	Anterolateral approach	9.91 <± 17.01>	2	4	3	4	90

^1^Identifying details have been anonymized for peer review

**Table 4:** Comparison of clinical studies on the DMC only with CICT

Study	Number of Patients	Mean age of patients in years (range)	Follow-up (months)	Comorbidities	Hip-function preoperative (range)	Hip-function postoperative (range)	Dislocation postoperative (%)	Loosening postoperative (%)	Infection postoperative (%)	Revisions postoperative (%)	Cup Survivorship (%)	Mean operation time (min)
Stambough [34]	8	60.6 (51–71)	34.8	–	–	–	2/8(25)	0	1/8 (12.5)	2/8 (25)	85	–
Evangelista [42]	18	62 (30–86)	36	–	46 (40–79)	65 (41–97)	0	0	1/16(6.25)	1/16 (6.25)	100	–
Chalmers [41]	18	64 (37–81)	36	–	HHS 47 (37–60)	HHS 81 (62–98)	3/18 (17)	0	0	2/18 (11.1)	92.3	–
Wegrzyn [40]	28	82 (74–93)	42	56/44% ASA 3/2	HHS 71 (69–74)	HHS 88 (82–95)	0	0	0	0	–	107
Moreta [43]	10	79.2 (71–87)	42	CCI 4.3	HHS 49.3(33–62)	HHS 71.3 (22–91)	1/10 (10)	0	0	1/10 (10)	92.2	–
Bellova [26]	33	78.6 (63–93)	30	ASA 2.72 CCI 5.1	–	HHS 59.4 (29–91), WOMAC 60	2/26 (7.7)	0	10	2/33 (6)	86.8	124
Saleh^1^	10	79.1 (66–93)	9.1	ASA 2.5	–	mHHS 60.89	2/10 (20)	1/10 (10)	2/10 (20)	3/10 (30)	90	102.80

*ASA* American Society of Anesthesiologists (score), *CCI* Charlson Comorbidity Index; *HHS* Harris Hip Score, *mHHS* modified Harris Hip Score; *min* minutes; *n* number; *WOMAC* Western Ontario and McMasters University Osteoarthritis (Index)

^1^Identifying details have been anonymized for peer review

We compared our results (regardless of which technique we used) with other studies, including only revision THA in those studies. Our findings revealed 4 patients with post-DMC operation hip dislocation, 2 of whom received a revision surgery. Additionally, post-DMC revision surgery was performed in 1 patient who developed an infection and another 1 who developed femoral stem loosening, amounting to a total of 4 revision surgeries (Table 2). The resulting cup survivorship post-DMC among the 20 patients was 90%. Table 3 illustrates a significant reduction in postoperative dislocation rates following THA, incorporating both our findings and data reported in several previous studies. These findings imply the advantages of using DMC as implants in revision THA.

The comparison between the two techniques (CICT and DIBT) revealed no significant difference in the outcome, as mentioned previously in Table 2. In our work with CICT (group 1), a slightly longer operation time than with the usual technique (group 2) was noticed. On the other hand, it has a shorter operation time than that reported by Wegrzyn et al. and Bellova et al. [26, 40]. We also noted a better outcome in terms of reducing the rate of postoperative dislocation in comparison to preoperative (Table 2). Out of the 10 patients 3 had postoperative revision due to either dislocation, infection or both.

In line with the findings of previous studies, we found favorable outcomes with the use of DMC as CICT technique in group 1 (Table 4). For example, postoperative dislocation proportion after CICT in our study was 20% similar to the findings of Stambough et al. (25%) [34] and Chalmers et al. (17 %) [41]. The cup survivorship in our study was 90%, which falls within the previously reported range (85–100%) [26, 34, 40–43]. It is worth noting that our sample included mostly nonelective revision THA among geriatric patients with multiple comorbidities.

In 2014 De Martino et al. examined the results of different studies on the use of DMC in primary and revision THA. The 5‑year survivorship of DMC was 94.5–98% with a dislocation rate of 1.1–5.5% [6].

After 3 years De Martino et al. published a systematic review of 59 articles evaluating the dislocation rate following THA after implantation of DMC. They divided a total of 17,908 THA into 2 categories: primary THA and revision THA. They reported a dislocation rate of 0.9% in the primary THA patients compared to 3.0% in the revision THA patients. The mean rate of hip dislocation was 0.7% in primary THA patients and 1.3% in revision THA patients [5]. Plummer et al. reported an improved HHS among patients who received revision THA following postoperative dislocation; 45 points preoperatively vs. 90 points postoperatively [44].

Jonker et al. compared the rate of dislocation between DMC and traditional unipolar cups in a systematic review in 2019. The study reported lower rates of hip dislocation and revision after implantation of DMC [45].

Another systematic review by Ciolli et al. including 3426 patients showed the results of cementation of DMC in primary (25.5% of the cases) as well as revision THA (74.5%). The estimated prevalence of overall postoperative dislocation was around 3%, with a cup survivorship of 93.5% [33].

A recent study on DMC by de Cano and Trias in 2023 examined the outcomes of THA revision via a cemented DMC. They divided the patients into 3 groups based on the technique: CICT in pre-existing acetabular cup, DMC in BSR or DIBT. The results revealed an improvement according to the modified disability (MD) scale from 8.34 preoperatively to 15.55 postoperatively [9]. It is worth noting the results of a systematic review highlighting the favorable outcomes of DMC compared to constrained cups. Among 5617 THAs the proportions of dislocation and loosening were lower in DMC (2.6% vs. 11.0%, and 1.0% vs. 2.0%, respectively). Additionally, the cup survival rate was higher in patients who underwent DMC [8]. In summary, all the mentioned studies demonstrated the positive effect of DMC in reducing the dislocation rate, not only in primary cases but also in revision cases.

Recently, some operators have introduced the cementation of DMC in a fixed acetabular cup as an off-label technique (CICT), especially in the management of recurrent hip dislocation. The aim was mainly to reduce intraoperative complications such as blood loss and bone defects as well as operation time. This is particularly useful for frail patients with high surgical risk [26]. We found 6 published studies discussing this technique as an alternative to the usual DMC implantation approach (Table 4). Wegrzyn et al. published a study that included 28 patients, who underwent only elective operations via CICT. They reported no cases of postoperative dislocation or dissociation with HHS 88 at a mean follow-up of 3.5 years [40]. Chalmers et al. and Moreta et al. also demonstrated better postoperative outcomes using CICT. Chalmers et al. reported only 3 cases of dislocation and no dissociation in a study that included 18 patients with HHS of 82 with a mean follow-up of 3 years [41]. Moreta et al. reported only 1 case of dislocation and no dissociation in a study that included 10 patients with HHS of 71 with a mean follow-up of 3.5 years [43].

A study by Bellova et al. in 2021 involving 33 patients, but unlike the other studies included also nonelective cases that amounted to nearly 40%. Bellova et al. reported only 2 patients with dislocation and 1 with dissociation with a mean surgery duration of 124 min [26]. Wegrzyn et al. reported CICT failure because of loosening between the cement and the pre-existing cup, and not between DMC and the cement layer. This was due to the lack of surface roughness or incorrect cementation between DMC and the fixed cup. Consequently, they recommended a cement layer with a thickness of 2–3 mm between the 2 cups and a size difference of 10 mm between the DMC and the outer diameter of the fixed acetabular cup [46]. In accordance with the recommendations of Wegrzyn et al. CICT is not suitable if the fixed acetabular cup is smaller than 54 mm [46]; however, Bellova et al. reported a thinner cement layer in approximately 65% of the cases, as the size of the fixed acetabular cup was smaller than 54 mm. This allows the usage of CICT technique with smaller fixed cup sizes (up to size 48 mm) [26]. Based on our observed results with DMC via CICT, we can consider it as a successful intervention on the short-term follow-up (Tables 2 and 4).

This study had various limitations: it was retrospective, included a small sample size, had short-term follow-up in some cases and involved different implant types; however, one strength of our study was that we included nonelective surgeries in multimorbid patients, unlike previous studies which focused on elective surgeries.

## Conclusion

Hip dislocation remains a challenging complication after primary or revision THA. The use of DMC improved the postoperative dislocation rate. The off-label implantation approach (CICT) can provide positive results, particularly in older and comorbid patients, by reducing bone injury, bleeding and in some cases, operative time during short-term follow-up. We did not observe any significant drawbacks when comparing both techniques (CICT and DIBT) in our study; however, we recommend further research with a bigger sample size and a longer follow-up time.

## Data Availability

The datasets used and/or analyzed during the current study are available from the corresponding author on reasonable request.

## Abbreviations

ASA: American Society of Anesthesiologists
BMI: Body mass index
CICT: Cup-in-cup technique
DIBT: Direct in bone technique
DMC: Dual mobility cup
THA: Total hip arthroplasty

## Declarations

Approval from the local institutional review board was obtained in view of the retrospective nature of the study, and all procedures performed were part of routine clinical care. This research was performed in accordance with the declaration of Helsinki. Consent to participate: All individuals have given general consent in the use of their data, including imaging, for analysis and publication. This has been approved by the Ethics Committee. Informed consent was obtained from all subjects and/or their legal guardian under Ethical approval and consent to participate section.
